# The Role of Cytomegalovirus-specific Immunoglobulins as Modulators of Antigen-specific T-cell Expansion In Vitro

**DOI:** 10.1097/TXD.0000000000001877

**Published:** 2025-11-07

**Authors:** Amina Abu-Omar, Saskia Bronder, Janine Mihm, David Schmit, Danilo Fliser, Urban Sester, Martina Sester, Tina Schmidt

**Affiliations:** 1 Department of Internal Medicine IV, Saarland University, Homburg, Germany.; 2 Department of Transplant and Infection Immunology, Saarland University, Campus Homburg, Germany.; 3 Department of Nephrology, Transplantation, and Immunology, Westpfalzklinikum Kaiserslautern, Kaiserslautern, Germany.; 4 Center for Gender-specific Biology and Medicine (CGBM), Saarland University, Homburg, Germany.

## Abstract

**Background.:**

Cytomegalovirus-specific immunoglobulins (CMV-IVIg) can contribute to viral control after transplantation. Apart from neutralizing antiviral activity, knowledge on their potential indirect effects on the restoration of virus-specific T-cell immunity is limited. Therefore, we tested whether CMV-IVIg may affect cytokine induction and proliferation of CMV-specific T cells in vitro.

**Methods.:**

Blood samples of 38 individuals (23 kidney transplant recipients and 15 immunocompetent controls) were stimulated using CMV antigens in saturating and low antigen concentration, or with the polyclonal stimulus *Staphylococcus aureus* enterotoxin B in the presence or absence of CMV-IVIg. CD4 and CD8 T-cell effector function, including the induction of cytokines interferon-gamma, tumor necrosis factor, and interleukin-2, was characterized after 6 h, and specific proliferation was quantified after 5 d using flow cytometry.

**Results.:**

Irrespective of antigen concentration, the presence or absence of CMV-IVIg had no effect on the percentage of CMV-specific CD4 and CD8 T cells producing interferon-gamma, tumor necrosis factor, or interleukin-2. However, proliferation of CMV-specific CD3 T cells, including CD4 and CD8 T-cell subpopulations, was significantly higher in the presence of CMV-IVIg at both saturating (*P* = 0.007) and low antigen concentrations (*P* = 0.022). In contrast, a lower percentage of both cytokine-producing T cells (*P* < 0.0001) and proliferating T cells (*P* < 0.0001) was observed in the presence of CMV-IVIg after polyclonal stimulation.

**Conclusions.:**

CMV-IVIg did not have any effect on immediate T-cell effector function. However, the marked effect of CMV-IVIg on increasing the proliferation of CMV-specific T cells while concomitantly reducing polyclonal T-cell function may have implications for the therapeutic use of immunoglobulins to restore CMV-specific T cells in patients with active CMV infection without increasing the alloreactive burden.

## INTRODUCTION

Primary infection or reactivation of cytomegalovirus (CMV) is one of the most common infectious complications after solid organ transplantation (SOT) due to systemic and lifelong immunosuppressive therapy. CMV management in SOT has improved considerably since the availability of antiviral drugs such as (val)ganciclovir, foscarnet, or, more recently, letermovir and maribavir.^1^ Nevertheless, CMV control after SOT can be difficult, and complications such as CMV end-organ disease, resistance, or drug side effects can impede successful treatment outcomes. In addition to drug therapy with direct antiviral activity, indirect therapeutic measures to strengthen antiviral immunity have been used and may be applied, including adjusting immunosuppression, and/or adjunct use of CMV-specific immunoglobulins (CMV-IVIg) or adoptive transfer of CMV-specific T cells.^1,2^

CMV-IVIg for therapeutic use are derived from pooled human plasma enriched for anti-CMV antibodies. CMV-IVIg are licensed for the prophylaxis of CMV infections and disease and have been used as an adjunct to antiviral drugs, in patients with hypogammaglobulinemia, or more rarely, as monotherapy in cases of intolerance or resistance to antiviral drugs.^1-4^ Thus, although CMV-IVIg are not generally recommended for use, some benefit has been demonstrated in these specific circumstances, especially in persons after thoracic transplantation.^1^ Immunoglobulins provide passive CMV-specific immunity by neutralizing circulating virus particles, thereby facilitating virus elimination by opsonization and phagocytosis.^5^ The titers of CMV-IgG have been shown to correlate with CMV neutralizing activities.^6,7^ Evidence from murine models have also suggested that protection from infection or reactivation with murine CMV may depend on the specificity of the immunoglobulins toward the infecting strain.^8^ Moreover, clinical observations suggest that CMV-IVIg may exert an immunomodulatory activity by increasing CMV-specific cellular immunity, a key factor in controlling CMV-related diseases and limiting viral replication.^9-11^ Finally, the immunomodulatory activity of CMV-IVIg or IVIg also seems to extend to protecting from allograft rejection by exerting immunosuppressive properties.^12,13^ However, the extent and the mechanism of this immunomodulatory function of CMV-IVIg products on CMV-specific cellular immunity and polyclonal cellular immunity are not yet fully understood. As a consequence, the need for a better understanding of the role of CMV-IVIg products to optimize the management of CMV complications was recently highlighted by the updated international consensus guidelines as an important area for future research.^1^

We therefore analyzed the effect of CMV-IVIg on the CMV-specific T cells in vitro. Blood samples from both kidney transplant recipients and healthy controls were used to compare cytokine induction as the immediate effector function of CMV-specific T cells in the presence or absence of clinically applied preparations of CMV-IVIg added in vitro. We applied this product in combination with whole blood samples without isolation of T cells to reflect the situation in vivo and to detect both direct and indirect effects of immunoglobulins on T cells. Moreover, the effect of CMV-IVIg on T-cell proliferation was analyzed. Finally, apart from the effect on CMV-specific T cells, we analyzed the effect of CMV-IVIg on polyclonal effector function and proliferation.

## MATERIALS AND METHODS

### Study Design and Participants

In this study, both healthy controls and kidney transplant recipients with known CMV serostatus were recruited at the Saarland University Medical Center. All individuals were adults older than 18 y. No formal sample size calculation was performed because of the exploratory nature of the study, but we aimed to include at least 15 individuals in each experiment. CMV prevention strategies applied at the time of transplantation included a history of preemptive therapy for CMV-seropositive recipients, and 3 mo of prophylaxis with valganciclovir in CMV-seronegative recipients of CMV-seropositive donors. CMV-seronegative recipients of CMV-seronegative donors did not receive any antiviral prophylaxis or preemptive therapy. Transplant recipients were at least 4 mo after transplantation, and neither had detectable CMV-DNAemia nor antiviral prophylaxis or therapy at the time of analysis. Clinical data on transplantation and therapeutic immunosuppression were collected from electronic medical records. A total of 4.7 mL of heparinized blood was drawn (in patients before intake of immunosuppressive drugs). The study was approved by the ethics committee of the Ärztekammer des Saarlandes (reference number 249/20), and written informed consent was obtained from all individuals.

### Characterization of CMV-specific and Polyclonal T-cell Effector Function

To determine the effect of CMV-IVIg on polyclonal versus CMV-specific T-cell effector function, activation, and cytokine induction was analyzed directly from heparinized whole blood without further isolation after in vitro stimulation with a CMV lysate in the presence and absence of CMV-IVIg. This procedure was chosen to reflect the situation in vivo. The CMV-IgG content of this CMV-IVIg product was approximately 17.5-fold enriched compared with the average CMV-IgG levels of healthy donors (see **Supplemental Digital Content Information, SDC,** https://links.lww.com/TXD/A806). CMV-specific T cells were stimulated using a lysate from CMV-infected fibroblasts or a control lysate from noninfected fibroblasts (Virion/Serion, Würzburg, Germany) as previously described^14^ at 2 different concentrations (32 μL/mL and 6.4 µL/mL blood). Stimulation with 2.5 µg/mL *Staphylococcus aureus* enterotoxin B (SEB, Sigma, Germany) served as CMV nonspecific polyclonal control. CMV-IVIg (Cytotect, Biotest, Dreieich, Germany) or H_2_O_dest_ were added at a final concentration of 2 µL/mL whole blood. Before T-cell stimulation, CMV-IVIg and stimulatory antigens were preincubated at 4–8 °C for 16–24 h to allow potential antigen-antibody complex formation. Thereafter, 450 µL of heparinized whole blood samples were stimulated for a total of 6 h with the antigen-immunoglobulin mix in the presence of costimulatory antibodies against CD28 and CD49d (clone L293 and clone 9F10, 1 μg/mL each). To accumulate cytokines intracellularly, 10 µg/mL Brefeldin A was added after 2 h, and samples were processed as previously described.^14^ After stimulation, the cells were immunostained using anti-CD4 (clone SK3, 1:33.3), anti-CD8 (clone SK1, 1:12.5), anti-CD69 (clone L78, 1:33.3), anti-interferon-gamma (IFN-γ; clone 4S.B3, 1:100), anti-interleukin (IL)2 (clone MQ1-1 7H12, 1:12.5), and anti–tumor necrosis factor (TNF; clone MAb11, 1:20), and analyzed using flow cytometry (BD FACS Canto II and FACSDiva software 6.1.3.). CMV-specific CD4 or CD8 T cells were identified by coexpression of CD69 and either IFN-γ, TNF, or IL2. The gating strategy is shown in **Figure S1** (**SDC,** https://links.lww.com/TXD/A806). Specific CD4 T-cell levels were quantified after subtraction of reactivity after negative control stimulation as previously established.^15^

### Analysis of CMV-specific T-cell Proliferation

CMV-specific T-cell proliferation of peripheral blood mononuclear cells (PBMCs) was analyzed using a carboxyfluorescein succinimidyl ester (CFSE) assay as described previously.^16^ All experiments were performed in the presence and absence of CMV-IVIg. Again, 2 concentrations of CMV lysate or control lysate (BioWhittaker, each 32 and 6.4 μL/mL, respectively), and 2.5 µg/mL SEB were used as stimuli. Stimulatory antigens were preincubated with CMV-IVIg or H_2_O_dest_ for 16–24 h before stimulation as described earlier. PBMCs were isolated by Ficoll density gradient (Linaris) and stained with CFSE (5 µM, Invitrogen) according to the manufacturer’s instructions. The cells were resuspended in Roswell Park Memorial Institute medium with 5% fetal calf serum and 1% antibiotics at a cell count of 2 × 10^7^ cells/mL. Stimulation was carried out in 96-well plates (600.000 PBMCs per well) at 37 °C and 5% CO_2_ for 5 d with the preincubated antigen-immunoglobulin mixes. SEB-stimulated cells were split 1:1 on day 3. Flow cytometric analysis was performed using antibodies toward CD3 (clone SK7, 1:50), CD4 (clone SK3, 1:12.5), and CD8 (clone SK1, 1:12.5). The gating strategy to quantify the percentage of proliferating CD3, CD4, and CD8 T cells is shown in **Figure S2** (**SDC,** https://links.lww.com/TXD/A806).

### Statistical Analysis

All statistical analyses were performed using GraphPad Prism 10.5.0 software (GraphPad, San Diego, CA) using 2-tailed tests. The Wilcoxon matched-pairs signed-rank test was used to compare paired data between 2 groups. A *P* value of <0.05 was considered statistically significant.

## RESULTS

### Participants

A total of 23 kidney transplant recipients (20 CMV seropositive, 3 CMV seronegative) and 15 healthy immunocompetent controls (13 CMV seropositive, 2 CMV seronegative) were recruited. Demographic characteristics, including age, sex, underlying disease that led to renal failure, time after transplantation, immunosuppressive drug regimens, and differential blood counts, are shown in Table 1. Kidney transplant recipients have been transplanted for a median of 5.4 y (interquartile range [IQR], 8.0 y). At the time of testing, kidney transplant recipients did not receive any antiviral therapy or prophylaxis, had no detectable CMV-DNAemia by polymerase chain reaction, and did not show any clinical signs of active CMV infection.

**TABLE 1.** Demographic and clinical characteristics of healthy controls and kidney transplant recipients

Characteristics	Controls (N = 15)	Transplant recipients (N = 23)
Age, y, median (IQR)	52.2 (20.8)	55.8 (16.2)
Sex, n (%)		
Female	11 (73.3%)	8 (34.8%)
Male	4 (26.7%)	15 (65.2%)
CMV serostatus, n (%)		
Positive	13 (86.7%)	20 (87.0%)
Negative	2 (13.3%)	3 (13.0%)
Years after transplantation, median (IQR)	NA	5.4 (8.0)^*a*^
CMV prevention strategy, n (%)		
None (D^–^/R^–^)	NA	2 (8.7%)
VGCV prophylaxis (D^+^/R^–^)^*b*^	NA	6 (26.1%)
Preemptive therapy (R^+^)	NA	15 (65.2%)
Differential blood counts,^*c*^ cells/µL, median (IQR)		
Leukocytes	6100 (2100)	6500 (3500)
Lymphocytes	2024 (538)	1606 (1238)
Monocytes	476 (209)	676 (299)
Granulocytes	3229 (1851)	4884 (2313)
Immunosuppressive drugs, n (%)	NA	
Steroids/CNI/MMF	NA	18 (78.26%)
CNI/MMF	NA	2 (8.70%)
Steroids/mTORi	NA	1 (4.35%)
Steroids/CNI	NA	1 (4.35%)
Unknown	NA	1 (4.35%)
Nephrological disease, n (%)	NA	NA
Congenital	NA	4 (17.39%)
Acquired	NA	15 (65.2%)
Autoimmune	NA	8
Secondary	NA	7
Unclear	NA	4 (17.39%)

^*a*^One patient within the first year, all other patients >1 y after transplantation.

^*b*^Prophylaxis was given for 3 mo.

^*c*^Differential blood counts from 2 transplant patients were missing.

CMV, cytomegalovirus; CNI, calcineurin inhibitor; IQR, interquartile range; MMF, mycophenolate mofetil; mTORi, mammalian target of rapamycin inhibitor; VGCV, valganciclovir.

### CMV-specific Cytokine Induction of CD4 and CD8 T cells in the Presence of CMV-IVIg

**FIGURE 1.**
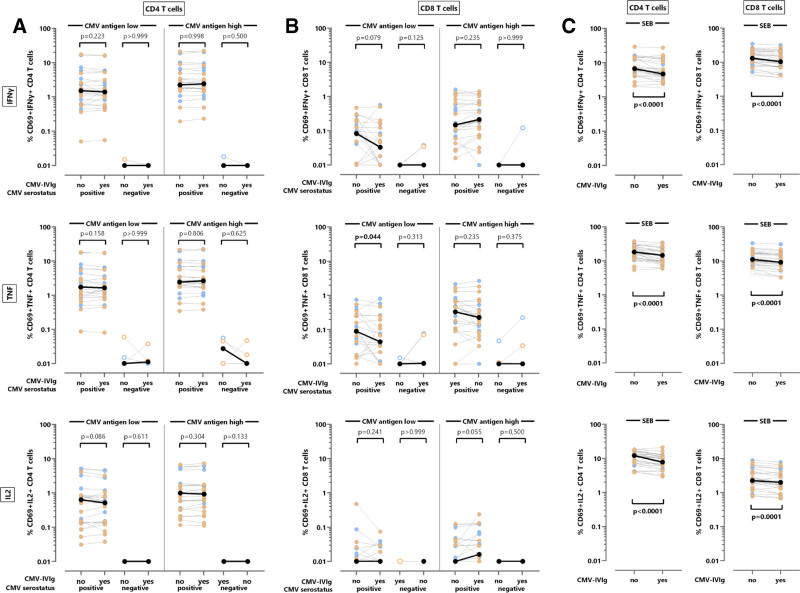
T-cell cytokine induction in the presence of CMV-specific immunoglobulins. CD4 T cells (A) or CD8 T cells (B) were analyzed in kidney transplant recipients (n = 18, orange symbols, 15 CMV seropositive [closed symbols], 3 CMV seronegative [open symbols]) and in healthy controls (n = 10, blue symbols, 8 CMV seropositive, 2 CMV seronegative) in the presence and absence of CMV-IVIg after stimulation with 2 different concentrations of CMV antigen lysate (32 µL/mL “high” (saturating) and 6.4 µL/mL “low”) and subtraction of antigen negative control. C, CD4 and CD8 T cells from all individuals were stimulated with SEB in the presence or absence of CMV-IVIg (n = 18 kidney transplant recipients and 10 healthy controls). Shown are the percentages of activated CD69-positive CD4 or CD8 T cells producing IFN-γ, TNF, or IL-2. Differences in the percentage of T cells in the presence and absence of CMV-IVIg were calculated using the Wilcoxon matched-pairs signed-rank test, with *P* values indicating significant differences highlighted in bold font, and fold changes shown in Table 2. The highlighted black symbols denote the medians. CMV-IVIg, CMV-specific immunoglobulin; IFN-γ, interferon-gamma; IL, interleukin; SEB, *Staphylococcus aureus* enterotoxin B; TNF, tumor necrosis factor.

CMV-specific effector T-cell function was characterized in a subset of 28 individuals (18 kidney transplant recipients, 10 healthy controls) after a 6-h stimulation in the presence and absence of CMV-IVIg, followed by intracellular cytokine staining. CMV lysate was used as a stimulus both at saturating concentrations (“high” 32 µL/mL) and at a 5-fold lower concentration (“low” 6.4 µL/mL). Likewise, similar concentrations of a control antigen were treated with CMV-IVIg and used as the respective negative control stimulus. In addition, SEB served as a polyclonal stimulus for T cells that is independent of antigen uptake, processing, and presentation. The percentage of reactive CD4 and CD8 T cells was determined on the basis of coexpression of CD69 with either IFN-γ, TNF, or IL-2, with the reactivity of the respective control antigens subtracted (Figure 1).

**TABLE 2.** Fold change in cytokine-producing and proliferating T-cell populations after CMV stimulation and polyclonal stimulation in the presence of CMV-IVIg

	CD4 T cells		CD8 T cells		CD3 T cells		CD4 T cells	CD8 T cells	CD3 T cells
	CMV antigen		CMV antigen		CMV antigen		SEB	SEB	SEB
	Low concentration^*a*^	High concentration^*a*^	Low concentration^*a*^	High concentration^*a*^	Low concentration^*a*^	High concentration^*a*^			
IFN-γ	0.97 (0.23) *P* = 0.223	1.01 (0.15) *P *= 0.988	0.90 (1.03) *P *= 0.079	1.09 (0.65) *P* = 0.226	ND	ND	**0.83 (0.21**) ***P* < 0.0001**	**0.88 (0.13**) ***P* < 0.0001**	ND
TNF	0.96 (0.21) *P* = 0.158	0.97 (0.15) *P* = 0.806	**0.78 (0.89**) ***P *= 0.044**	1.10 (0.88) *P* = 0.235	ND	ND	**0.80 (0.21**) ***P* < 0.0001**	**0.88 (0.10**) ***P* < 0.0001**	ND
IL-2	0.96 (0.30) *P* = 0.086	1.02 (0.14) *P* = 0.304	1.00 (0.17) *P *= 0.241	1.05 (0.25) *P* = 0.055	ND	ND	**0.77 (0.23**) ***P* < 0.0001**	**0.86 (0.19**) ***P* = 0.0001**	ND
Proliferation	**2.00 (1.45**) ***P* = 0.022**	**1.17 (0.72**) ***P *= 0.007**	**2.64 (3.70**) ***P *= 0.017**	1.49 (2.36) *P *= 0.314	**1.82 (1.75**) ***P* = 0.022**	**1.19 (0.87**) ***P* = 0.007**	**0.90 (0.14**) ***P* < 0.0001**	**0.95 (0.22**) ***P* = 0.0004**	**0.92 (0.14**) ***P* < 0.0001**

Values refer to the fold change (interquartile range) and *P* value in the percentage of cytokine-producing CD4 and CD8 T cells or in the percentage of proliferating CD3, CD4, and CD8 T cells. Results with significant changes in the presence of CMV-IVIg based on the Wilcoxon matched-pairs signed-rank test are shown in bold font.

^*a*^Two concentrations of CMV antigen lysate were used (32 µL/mL “high” (saturating) and 6.4 µL/mL “low”).

CMV-IVIg, CMV-specific immunoglobulin; IFN-γ, interferon-gamma; IL-2, interleukin 2; ND, not determined; TNF, tumor necrosis factor.

All CMV-seropositive individuals (15 kidney transplant recipients and 8 controls, distinguished by orange and blue symbols, respectively) had detectable cytokine-producing CMV-specific CD4 T cells. The addition of CMV-IVIg had no modulatory effect on the induction of IFN-γ, TNF, or IL-2 after specific stimulation at either the high or the low concentration of CMV lysate (Figure 1A; fold changes shown in Table 2). CMV-specific CD8 T-cell levels were generally lower. Apart from a minor effect on TNF and IL-2, their cytokine induction remained largely unaffected by the presence of CMV-IVIg (Figure 1B). We also included CMV-seronegative individuals to assess the potential effects of CMV-IVIg on T cells after CMV-specific stimulation. As expected, no relevant CMV-specific induction of cytokines was detected among CMV seronegative individuals (n = 5). Their IFN-γ and IL-2 levels were low, whereas TNF levels were slightly higher. Moreover, CMV-IVIg did not have any modulatory activity on their cytokine expression (Figure 1A and B).

Unlike CMV-specific T cells, the percentage of CD4 and CD8 T cells producing IFN-γ, TNF, or IL-2 after polyclonal stimulation with SEB was significantly reduced in the presence of CMV-IVIg (Figure 1C; *P* < 0.0001), which is in line with the general immunosuppressive properties of IVIg. This decrease was observed for all tested populations and ranged from a 0.77-fold (IQR, 0.23) reduction in the percentage of IL-2-producing CD4 T cells to a 0.88-fold (IQR, 0.13) reduction in the percentage of IFN-γ-producing CD8 T cells (Table 2).

### CMV-specific T-cell Proliferation in the Presence and Absence of CMV-IVIg

**FIGURE 2.**
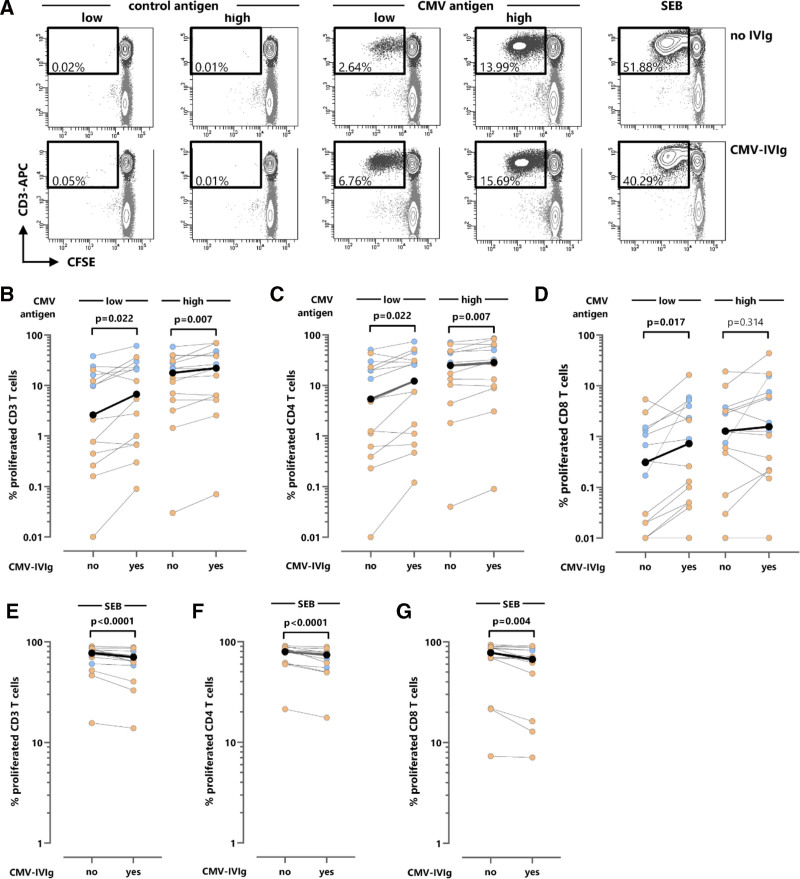
T-cell proliferation depending on the presence of CMV-specific immunoglobulins. A, Typical dot plots of the proliferation of CD3 T cells using a CFSE-based assay with blood from a kidney transplant recipient stimulated with 2 different concentrations of CMV lysate (32 µL/mL “high” (saturating) and 6.4 µL/mL “low”), respective control lysates, and SEB in the presence and absence of CMV-IVIg. Proliferating cells were quantified as a percentage of CFSE-low CD3 T cells. Results are shown for CMV-specific CD3 T cells (B), CD4 T cells (C), and CD8 T cells (D), and polyclonally stimulated CD3 T cells (E), CD4 T cells (F), and CD8 T cells (G) after stimulation of cells from CMV-seropositive kidney transplant recipients (n = 10, orange symbols) and healthy controls (n = 5, blue symbols) with the 2 different concentrations of CMV lysate or SEB in the presence and absence of CMV-IVIg. CMV-specific proliferation was quantified after subtraction of the respective negative control proliferation. Differences in the percentage of proliferating T cells in the presence and absence of CMV-IVIg were calculated using the Wilcoxon matched-pairs signed-rank test, with *P* values indicating significant differences highlighted in bold font, and fold changes shown in Table 2. The highlighted black symbols denote the medians. CFSE, carboxyfluorescein succinimidyl ester; CMV, cytomegalovirus; CMV-IVIg, CMV-specific immunoglobulin; SEB, *Staphylococcus aureus* enterotoxin B.

Apart from analyses of the immediate effector function of CMV-specific T cells, the proliferation of CD4 and CD8 T cells was evaluated in a subset of 15 CMV-seropositive individuals (10 kidney transplant recipients, 5 immunocompetent controls) after stimulation of PBMCs with saturating and low concentrations of CMV lysate (high and low, respectively) in the presence and absence of CMV-IVIg, respectively. Respective control lysates, in the presence or absence of CMV-IVIg, were used as negative controls, and SEB served as a control for CMV nonspecific proliferation. As shown in typical dotplots of proliferating CD3 T cells of a kidney transplant recipient (Figure 2A), control antigens did not elicit any relevant proliferation irrespective of the presence of CMV-IVIg. In contrast, the specific proliferation in response to both low and high CMV antigen concentrations was further increased in the presence of CMV-IVIg. Results from all individuals are shown in Figure 2B. Overall, the percentage of CMV-specific proliferating cells was higher among controls than among patients. Nevertheless, the proliferation of CMV-specific CD3 T cells from both groups showed a significant increase in the presence of CMV-IVIg, which was more pronounced after stimulation at low antigen concentration (1.88 [IQR, 1.71] fold increase, *P* = 0.022) than at saturating CMV antigen concentrations (1.19 [IQR, 0.87] fold increase, *P* = 0.007; Figure 2B; Table 2). The same held true for the proliferation of the CMV-specific CD4 and CD8 T-cell subpopulations (Figure 2C and D), except for CD8 T cells at saturating antigen concentration, where no further increase in proliferation was observed (Figure 2D).

As shown from the dotplots of CD3 T cells after polyclonal SEB stimulation, proliferation decreased in the presence of CMV-IVIg (Figure 2A). This decreasing effect of CMV-IVIg was not only observed for SEB-reactive CD3 T cells (0.92 [IQR, 0.14] fold decrease, *P* < 0.0001; Figure 2E; Table 2) but also for the respective CD4 and CD8 T-cell subpopulations after polyclonal stimulation (0.90 [IQR, 0.14] and 0.94 [IQR, 0.22] fold decrease, *P* < 0.0001 and *P* = 0.004, respectively; Figure 2F and G; Table 2). Thus, as with effector T cells producing cytokines, the presence of CMV-IVIg led to a reduction in the proliferation of polyclonal T cells.

## DISCUSSION

The risk of primary infection or reactivation with human CMV is increased after SOT due to the systemic and lifelong immunosuppression. CMV-IVIg have been used after SOT either as an adjunct to antiviral drugs or as monotherapy in cases of intolerance or resistance to antiviral drugs.^9-11^ The virus-neutralizing effect of CMV-IVIg is well known and may contribute to the inhibition and control of CMV replication. However, the indirect effect of CMV-IVIg on modulating CMV-specific T-cell responses, which is a key factor in controlling CMV-related disease and limiting viral replication, is poorly understood.^10,17,18^ In our in vitro study, we show a significant increase in CMV-specific T-cell proliferation in the presence of CMV-IVIg, whereas immunoglobulins did not have any direct effect on CMV-specific T-cell activation and cytokine induction after short-term stimulation. Interestingly, increased proliferation was observed only among CMV-specific T cells and contrasted with polyclonal T cells after stimulation with SEB, where the presence of CMV-IVIg led to a reduction in cytokine induction and proliferation. Thus, although both cytokine induction and proliferation of polyclonal T cells were suppressed, CMV-IVIg exerted a specific immunomodulatory function by increasing CMV-specific T-cell proliferation.

CMV-IVIg are used as an alternative prevention strategy in patients intolerant to (val)ganciclovir or in complicated CMV infections,^10^ and/or as an adjunct therapeutic regimen in patients with refractory or resistant CMV infection.^9,11^ In this situation, CMV-IVIg have been shown to contribute to a reduction in viral replication and induction of CMV-specific T-cell immunity in patients in vivo.^3,9,11,12^ However, controlled assessment of their direct effects on a patients’ specific T-cell response is difficult in vivo, as CMV-IVIg are usually applied in combination with antiviral and immunosuppressive drugs, the dosage and composition of which are further adjusted in the face of an active CMV infection. In this study, we used an in vitro system of stimulated T cells from immunosuppressed kidney transplant patients and healthy controls to more specifically test the immunomodulatory effect of CMV-IVIg on the specific T-cell response. We chose a clinically used product of immunoglobulins to closely reflect the situation in vivo. This product was enriched for CMV-IgG by approximately 17.5-fold as compared with healthy donors. In line with observations in vivo, we show that the proliferation of CMV-specific CD4 and CD8 T cells was induced in the presence of CMV-IVIg both after stimulation with CMV antigen at saturating and reduced antigen levels. Data from a recent study using PBMC from healthy controls suggested that CMV-IVIg may induce a low-level global stimulatory effect on cells independent of CMV serostatus. However, the number of tested individuals was low, and this reactivity was much lower compared with CMV-specific responses.^19^ We did not detect any effect of CMV-IVIg on immediate effector function, such as activation and cytokine induction, after CMV-specific stimulation times as short as 6 h. This is supported by the above-mentioned study performed from isolated PBMCs, where the percentage of cytokine-producing CD4 or CD8 T cells after CMV-specific stimulation did not differ in the presence or absence of CMV-IVIg.^19^ Together, this illustrates that these indirect effects of CMV-IVIg may require longer incubation times to unfold their effects in enhancing cytokine induction and proliferation of CMV-specific CD4 and CD8 T cells. The precise mode of action whereby proliferation is increased is currently unclear, but it may be speculated that specific immune complexes of CMV antigens opsonized with CMV-IVIg are better taken up by antigen-presenting cells.^5^ This better Fc receptor-mediated uptake of CMV antigens may lead to an increase in antigen presentation and to a better induction of T-cell effector function, including cytokine induction and proliferation in the long term. It is interesting to note that proliferation was more strongly induced by CMV-IVIg in the presence of nonsaturating concentrations of antigen, which may allow unmasking the specific effect of CMV-IVIg. Likewise, preliminary evidence suggests that immunoglobulin preparations with lower levels of CMV immunoglobulins exert a similar, albeit less pronounced effect on CMV-specific proliferation. Unlike the increase in CMV-specific proliferation, we show that the presence of CMV-IVIg was associated with decreased cytokine induction and proliferation of polyclonally stimulated CD4 and CD8 T cells, which has also been described before as a general feature of both CMV-IVIg and IVIg in vitro.^20,21^ Apart from suppressing polyclonal proliferation, IVIg have been shown to inhibit allogeneic T-cell proliferation,^22,23^ modulate dendritic cell function,^23^ induce B-cell hyporesponsiveness, block various activating receptors, and expand regulatory T cells.^5,13^ These contrasting properties of CMV-IVIg, which increase CMV-specific T-cell expansion while concomitantly decreasing polyclonal T-cell expansion, may be beneficial in the prevention and treatment of rejection episodes, as supported by observations in transplant recipients in vivo.^24-29^

Our study design has some inherent limitations, as the results of the in vitro study are not directly transferable to patients in vivo, and we cannot mechanistically distinguish between direct and indirect effects of CMV-IVIg or other components in the product on T cells. Nevertheless, our results are well in line with clinical observations, which, on one hand, show an increase in CMV-specific T cells in patients and control of viral replication upon receipt of adjunct CMV-IVIg,^9-11^ and on the other hand, support a contribution of CMV-IVIg toward preventing or treating rejection episodes.^24-27^ In addition, the limited sample size of subgroups did not allow for a direct comparison of patients and controls. Nevertheless, our sample size seems sufficient to reveal robust results, which were consistent over several tested cytokines and cell populations, and overall similar in both patients and controls. A further strength of the study is that both immunocompetent individuals and immunocompromised patients on maintenance immunosuppression were included. As patients were enrolled at a median of 5.4 y (IQR, 8.0 y) after transplantation, our in vitro experiments were performed without influencing factors such as antiviral drugs, detectable CMV-DNA, other coinfections, or variabilities in immunosuppression.

Further in vitro studies should address a more detailed analysis of the individual components of the immunoglobulin preparation, including the role of CMV-specific versus nonspecific IgG for inducing CMV-specific proliferation and reducing polyclonal proliferation. Moreover, the role of other blood cell populations, such as antigen-presenting cells or natural killer cells, should be explored to identify indirect regulatory mechanisms mediated by CMV-IVIg. Finally, in vivo randomized trials in CMV-seropositive patients managed in a preemptive setting, in the presence or absence of CMV-IVIg and/or IVIg, are necessary to assess their role in CMV-specific T-cell expansion without concomitantly inducing T cells toward other specificities. Similarly, randomized in vivo studies using combination therapy of antiviral drugs such as (val)ganciclovir, foscarnet, or maribavir with or without CMV-IVIg and/or IVIg would allow assessing the role of immunoglobulins in inducing CMV-specific T cells, thereby potentially reducing relapse rates in patients with refractory/resistant CMV infection. As recently recommended by the latest consensus guidelines,^1^ this type of study design would also allow evaluation of CMV-specific T-cell immunomonitoring in guiding the duration of these therapeutic interventions or secondary prophylaxis.

In conclusion, we showed that while CMV-IVIg has no effect on immediate effector function, the presence of immunoglobulins increases the proliferative activity of CMV-specific T cells and inhibits polyclonal T-cell activation and proliferation. These in vitro data provide novel insights into the role of CMV-IVIg in contributing to the induction of CMV-specific T cells in patients with active infections. Future studies, as outlined earlier, should investigate the mechanistic details underlying the direct and indirect effects of CMV-IVIg on inducing CMV-specific proliferation while concomitantly reducing polyclonal T-cell expansion. In addition, clinical studies are needed to confirm these immunomodulatory effects in vivo.

A.A.-O. and T.S. have received travel grant support from Biotest. M.S. has received support for this investigator-initiated study to the organization Saarland University by Biotest, and for investigator-initiated studies by Takeda and Astellas outside of the submitted work to the organization Saarland University, and honoraria for lectures from Biotest, Novartis, Takeda, MSD, and for participation in advisory boards from Biotest, Moderna, MSD, and Takeda. The other authors declare no conflicts of interest.

## ACKNOWLEDGMENTS

The authors acknowledge the technical support of Rebecca Urschel and Candida Guckelmus.

## Supplementary Material
