# Supplementary material for: The Role of Cytomegalovirus-specific Immunoglobulins as Modulators of Antigen-specific T-cell Expansion In Vitro
Source: Transplant Direct. 2025 Nov 7;11(12):e1877. doi: 10.1097/TXD.0000000000001877 (PMC12594292; doi:10.1097/TXD.0000000000001877)
Supplement: Supplementary file 1 [file txd-11-e1877-s001.pdf]

Supplementary digital content

The role of cytomegalovirus-specific immunoglobulins as modulators of antigen-specific T-cell expansion in vitro

Amina Abu-Omar, Saskia Bronder, MSc, Janine Mihm, MD, David Schmit, MD, Danilo Fliser, MD, Urban Sester, MD, Martina Sester, PhD and Tina Schmidt, PhD

This supplement contains

Supplementary information on the CMV-IVIg product

Figure S1

Figure S2

Supplementary digital content information

Supplementary information on the CMV-IVIg product

To estimate the extent of enrichment of CMV-IgG in our CMV-IVIg product that we used in our experiments (Cytotect, Biotest, Dreieich, Germany), an ELISA assay was used to quantify CMV-specific IgG (CMV IgG ELISA, Euroimmun, Lübeck, Germany). The product had a CMV-IgG level of 2778 RU/ml. As a comparison, the median CMV-IgG levels of a random cohort of 175 immunocompetent blood donors (mean 45.3±17.4 years of age) tested with the same assay was 159 (IQR 84) RU/ml. This is in the same range as the CMV-IgG level of an IVIg product not enriched for CMV-IgG (Varitect, VZV-IVIg, Biotest Dreieich, Germany), which was 161.06 RU/ml. Thus, this corresponds to an approximately 17.5-fold enrichment of CMV-IgG in the CMV-IVIg product as compared to average CMV-IgG levels of healthy donors or other immunoglobulin products.

Supplementary digital content figures

Figure S1

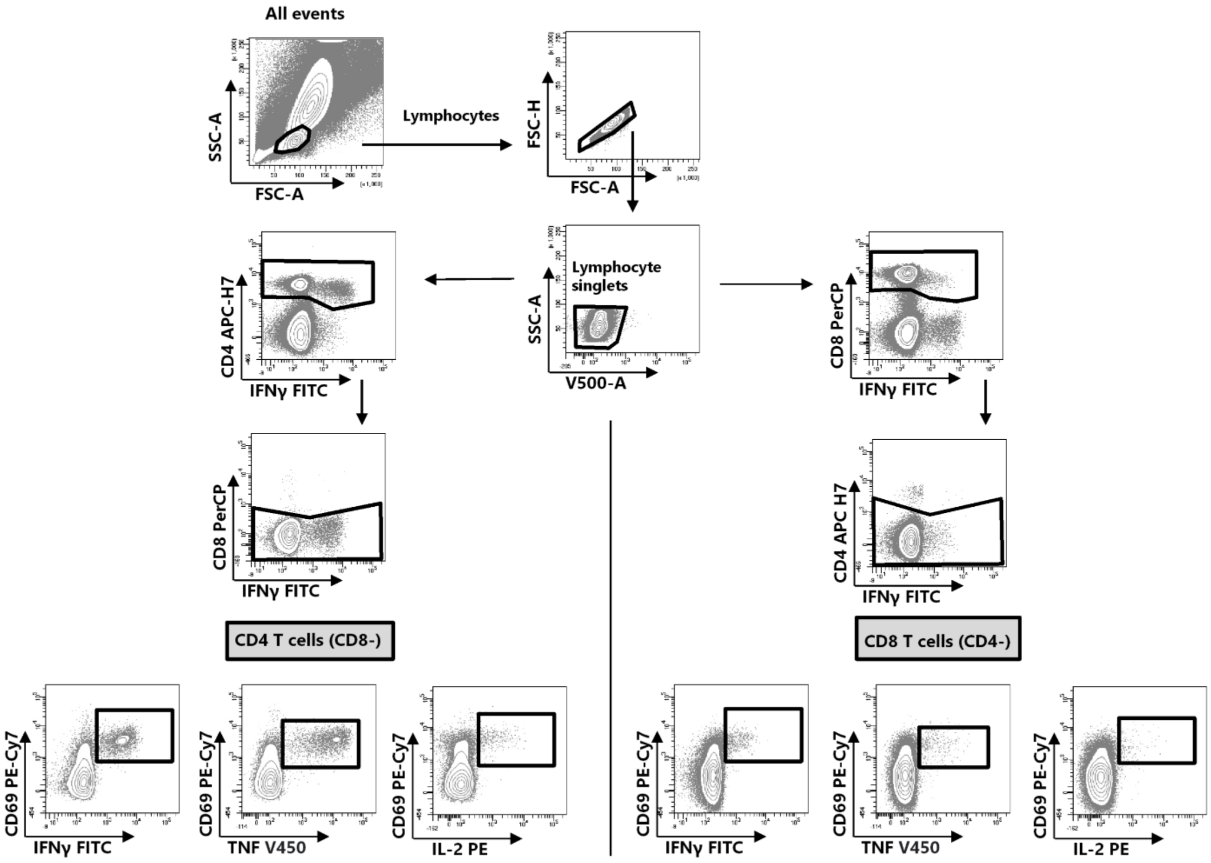

Figure S1: Gating strategy to identify CMV- or SEB-reactive CD4 and CD8 T cells co-expressing CD69 and IFNγ, TNF or IL2 after stimulation. Lymphocytes were identified among total events by backgating of CD4- and/or CD8-positive cells combined with signals for size (FSC) and granularity (SSC). Height and width area signals of FSC were used to exclude doublets. The gating strategy and boxes that were used to identify CD4 T cells (left side) or CD8 T cells (right side) co-expressing the activation marker CD69 and the cytokines IFNγ, TNF or IL2 are shown.

Figure S2

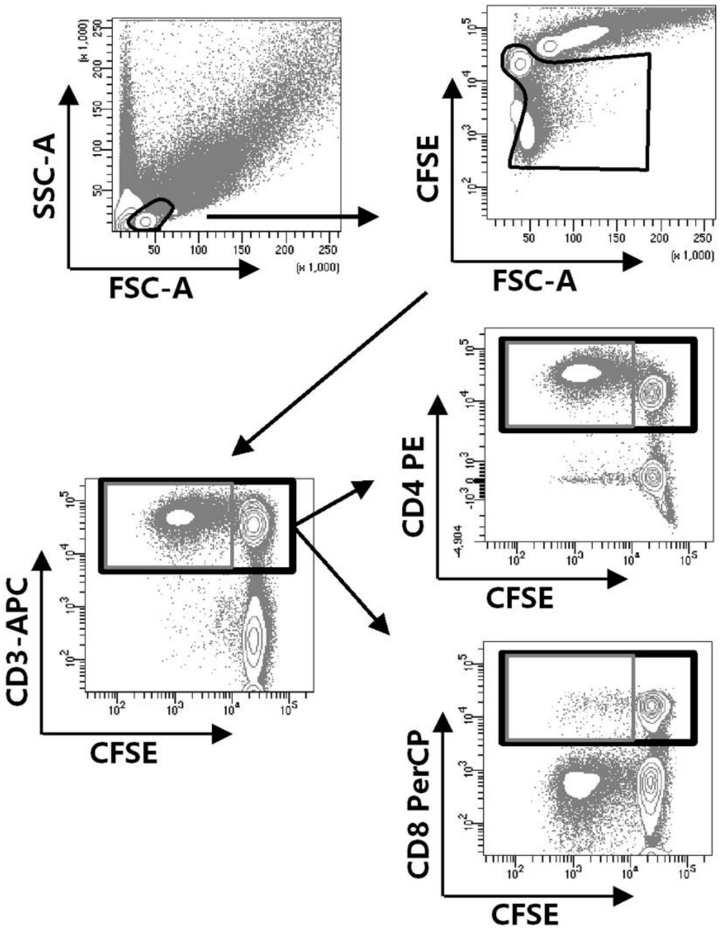

Figure S2: Gating strategy to identify the proliferating T cells after stimulation with CMV antigens or SEB. Lymphocytes were identified among total events by size (FSC) and granularity (SSC). Lymphocytes including CFSE-low cells of large size (FSC) were gated. This population was further separated based on expression of CD3 (all T cells) and further subdivided in CD4 and CD8 T cells. Gates show the whole population of CD3 T cells, CD4 T cells and CD8 T cells. Proliferating cells among the whole populations were identified as CFSE-low cells.
